# Assessing Command-Following and Communication With Vibro-Tactile P300 Brain-Computer Interface Tools in Patients With Unresponsive Wakefulness Syndrome

**DOI:** 10.3389/fnins.2018.00423

**Published:** 2018-06-29

**Authors:** Christoph Guger, Rossella Spataro, Frederic Pellas, Brendan Z. Allison, Alexander Heilinger, Rupert Ortner, Woosang Cho, Ren Xu, Vincenzo La Bella, Günter Edlinger, Jitka Annen, Giorgio Mandalá, Camille Chatelle, Steven Laureys

**Affiliations:** ^1^Guger Technologies OG, Graz, Austria; ^2^g.tec Medical Engineering GmbH, Schiedlberg, Austria; ^3^IRCCS Centro Neurolesi Bonino Pulejo, Palermo, Italy; ^4^ALS Clinical Research Center, BioNeC, University of Palermo, Palermo, Italy; ^5^Post-ICU Neurorehabilitation Unit, University Hospital of Nîmes, Nîmes, France; ^6^Department of Cognitive Science, University of California, San Diego, San Diego, CA, United States; ^7^Coma Science Group, GIGA Consciousness, University of Liège, Liège, Belgium; ^8^Rehabilitation Unit, Buccheri La Ferla Hospital, Palermo, Italy

**Keywords:** communication, unresponsive wakefulness syndrome, vegetative state, brain computer interface, evoked potentials, vibro-tactile P300

## Abstract

Persons diagnosed with disorders of consciousness (DOC) typically suffer from motor disablities, and thus assessing their spared cognitive abilities can be difficult. Recent research from several groups has shown that non-invasive brain-computer interface (BCI) technology can provide assessments of these patients' cognitive function that can supplement information provided through conventional behavioral assessment methods. In rare cases, BCIs may provide a binary communication mechanism. Here, we present results from a vibrotactile BCI assessment aiming at detecting command-following and communication in 12 unresponsive wakefulness syndrome (UWS) patients. Two different paradigms were administered at least once for every patient: (i) VT2 with two vibro-tactile stimulators fixed on the patient's left and right wrists and (ii) VT3 with three vibro-tactile stimulators fixed on both wrists and on the back. The patients were instructed to mentally count either the stimuli on the left or right wrist, which may elicit a robust P300 for the target wrist only. The EEG data from −100 to +600 ms around each stimulus were extracted and sub-divided into 8 data segments. This data was classified with linear discriminant analysis (using a 10 × 10 cross validation) and used to calibrate a BCI to assess command following and YES/NO communication abilities. The grand average VT2 accuracy across all patients was 38.3%, and the VT3 accuracy was 26.3%. Two patients achieved VT3 accuracy ≥80% and went through communication testing. One of these patients answered 4 out of 5 questions correctly in session 1, whereas the other patient answered 6/10 and 7/10 questions correctly in sessions 2 and 4. In 6 other patients, the VT2 or VT3 accuracy was above the significance threshold of 23% for at least one run, while in 4 patients, the accuracy was always below this threshold. The study highlights the importance of repeating EEG assessments to increase the chance of detecting command-following in patients with severe brain injury. Furthermore, the study shows that BCI technology can test command following in chronic UWS patients and can allow some of these patients to answer YES/NO questions.

## Introduction

Assessing consciousness and communication in persons with disorders of consciousness (DOC) is difficult. The current gold-standard is based on bedside observation of the patients' responses, but these patients may lack the ability to perform voluntary motor responses at the bedside. Standardized scales such as the Coma-Recovery-Scale-revised (CRS-R; Giacino et al., 2004) have been developed, but these tools are highly dependent on the patient's motor abilities. This dependence may prevent the detection of signs of consciousness or the possibility of communication in this population (Monti et al., 2010; Giacino et al., 2012; Risetti et al., 2013; Gibson et al., 2014; Ortner et al., 2017), and therefore also limit the diagnosis of some patients with locked in syndrome (LIS; i.e., paralyzed with remaining vertical eye movement control but conscious with preserved cognitive abilities; Patterson and Grabois, 1986).

Brain-computer interfaces (BCIs) were originally developed to establish a communication channel with LIS patients via brain activity alone, usually by measuring and analyzing the electroencephalographic (EEG) response for applications such as selecting letters (Wolpaw et al., 2002; Wolpaw and Wolpaw, 2012; Nam et al., 2018). Such BCIs have been validated with different types of EEG paradigms, including motor imagery (MI) (Guger et al., 2003; Acqualagna et al., 2016), steady-state visual evoked potentials (SSVEPs; Bin et al., 2009; Ahn et al., 2016) or P300 event-related potentials (ERPs; Guger et al., 2009, 2016; Lugo et al., 2014). The P300 may be elicited if an unlikely event occurs that is embedded in frequent events. P300 based-BCIs have been used widely due to several appealing features, including a short calibration time, robustness, and ease of use (Fazel-Rezai et al., 2012). Over the last decade, such BCIs have been developed using visual, auditory (Risetti et al., 2013; Rutkowski, 2016) or vibrotactile stimuli (Lugo et al., 2014; Gibson et al., 2016). A vibro-tactile P300 study with LIS patients showed that the BCI system can still extract information from the EEG, even if visual inspection of the averaged ERPs suggests this is impossible. This is because the EEG data from each single trial was analyzed using linear discriminate analysis (LDA), in contrast visual inspection of averaged ERPs (Lugo et al., 2014).

Vibro-tactile P300 testing has also been used with LIS/CLIS patients and healthy subjects, where the participant is asked to count a target (rare) tactile stimuli either on the right or left hand to answer YES/NO questions. Using this technique, healthy subjects without prior training achieved high accuracies and were able to communicate (Allison et al., 2017; Guger et al., 2017b). 12 LIS/CLIS patients achieved a mean accuracy of 76.6% in VT2 (vibro-tactile paradigm with 2 stimulators), 63.1% in VT3 (vibro-tactile paradigm with 3 stimulators), and 58.2% in MI modes after 1–2 training runs. 9 out of 12 LIS patients could communicate by using the vibro-tactile P300 paradigms (answering 8 out of 10 questions correctly on average) and 3 out of 12 could communicate with the MI paradigm (answering 4.7 out of 5 questions correctly on average). In previous work using vibrotactile P300 BCIs for LIS patients, 6 LIS patients attained a mean accuracy of 80% in a paradigm with 2 tactile stimulators (left and right hand) and 55.3% in a paradigm with 3 tactile stimulators (left and right hand, neck) (Lugo et al., 2014). In both paradigms, chance accuracy was 12.5%, and the results were statistically significant. Recently, a system using functional near infrared spectroscopy was used for communication with CLIS patients and patients entering CLIS in more than 40 sessions (Chaudhary et al., 2017).

BCIs are also of growing interest for the DOC population, as they may provide an online assessment of the patient's cognitive abilities when motor impairments prevent the patient from showing voluntary signs of consciousness at bedside (Guger et al., 2014, 2017a; Real et al., 2016; Chennu et al., 2017; Nam et al., 2018). This approach could be easily implemented in a clinical setting to supplement the behavioral diagnosis and decrease potential misdiagnosis, as shown in previous studies using active tasks (e.g., Monti et al., 2010; Cruse et al., 2011).

The current study uses vibro-tactile P300 tests with 2 (VT2) and 3 (VT3) tactors for the assessment of remaining brain response (classic oddball paradigm using 2 tactors) and command following with binary communication testing (active task using 3 tactors). The BCI classification accuracy and evoked potentials from the VT2 and VT3 paradigms are evaluated. We also aimed to assess the necessary classification accuracy for communication in unresponsive wakefulness syndrome (UWS) patients, and we investigated whether repeated assessments yield better results.

## Methods

### Participants

Patients were recruited by the University of Palermo, Italy. Inclusion criteria were age >18 years and clinical diagnosis of UWS (awakening without any volitional response at the bedside examination), irrespective of delay from disease onset and etiology.

The clinical definition of UWS was based on the repetitive administration (at least five times) of the Italian version of the CRS-R scale (Lombardi et al., 2007). The patients had no history of neurologic disorder prior to coma. The mechanical ventilation did not interfere with the EEG recordings because we used active EEG electrodes.

Ethical approval was available from the Ethical Committee Palermo from the University Hospital of Palermo. Written informed consent was obtained from a legal guardian. Measurements were performed by a medical doctor who was trained on the proper handling of the system.

A convenience sample of 12 patients enrolled in the study (12 UWS, 9 men; median age: 53.3 years, range: 19–91 years; time since injury: 1–28 months, median: 2 months) as shown in Table 1. The etiologies of the patients were: traumatic brain injury (*n* = 4), stroke (*n* = 2), hypoxia-ischemia brain injury (*n* = 4), subdural hematoma (*n* = 1), and meningoencephalitis (*n* = 1).

**Table 1:** Overview of patients participating in this study.

**Clinical state and #**	**Age range (years)**	**Etiology**	**Disease duration at first session (months)**	**Mechanical ventilation**	**CRS-R score on day of first BCI assessment**
UWS1	18-20	TBI	28	No	6
UWS2	18-20	TBI	9	No	6
UWS3	31-40	TBI	2	No	3
UWS4	31-40	HBI	9	No	6
UWS5	91-100	Stroke	1	No	6
UWS6	81-90	SDH	2	Yes	5
UWS7	61-70	ME	2	No	6
UWS8	51-60	HBI	1	Yes	4
UWS9	61-70	HBI	2	Yes	6
UWS10	71-80	HBI	1	Yes	5
UWS11	71-80	Stroke	1	No	6
UWS12	21-20	TBI	2	No	8

*TBI, Traumatic Brain Injury; HBI, Hypoxia-Ischemia Brain Injury; SDH, Subdural Hematoma; ME, Meningoencefalitis; BT, Brain Trauma; UWS, Unresponsive Wakefulness Syndrome; The age is given as a range to avoid indirectly identifiable patient data*.

### Materials

All data were acquired with the mindBEAGLE prototype (g.tec Guger Technologies OG, Austria). The system consists of active gel-based EEG electrodes connected to a biosignal amplifier (g.USBamp, g.tec) with 24 Bit resolution and a high oversampling rate to increase the signal to noise ratio of the data. The amplifier sends the EEG data via USB at 256 Hz to a computer system that runs the experimental paradigm in real-time. The system also presents the EEG data on a monitor for quality inspection, stores the data in floating point format for off-line processing, performs the real-time signal processing and manages all stimulus presentation.

The acquired EEG data are bandpass filtered between 0.1 and 30 Hz to remove baseline shifts and eliminate most EMG artifacts. The EEG electrodes used for the experiments were positioned at sites Fz, C3, Cz, C4, CP1, CPz, CP2, and Pz according to the extended International 10–20 System. The reference electrode was fixed on the right earlobe and the ground electrode was mounted on the forehead.

### Behavioral assessment

The CRS-R was administered after careful neurologic examination by trained neurologists (R.S., V.L.B.), about 30 min before the first BCI session. Patients were assessed when free of sedation for at least 24 h. Table 1 presents the resulting scores.

### BCI assessment

**Figure 1:**
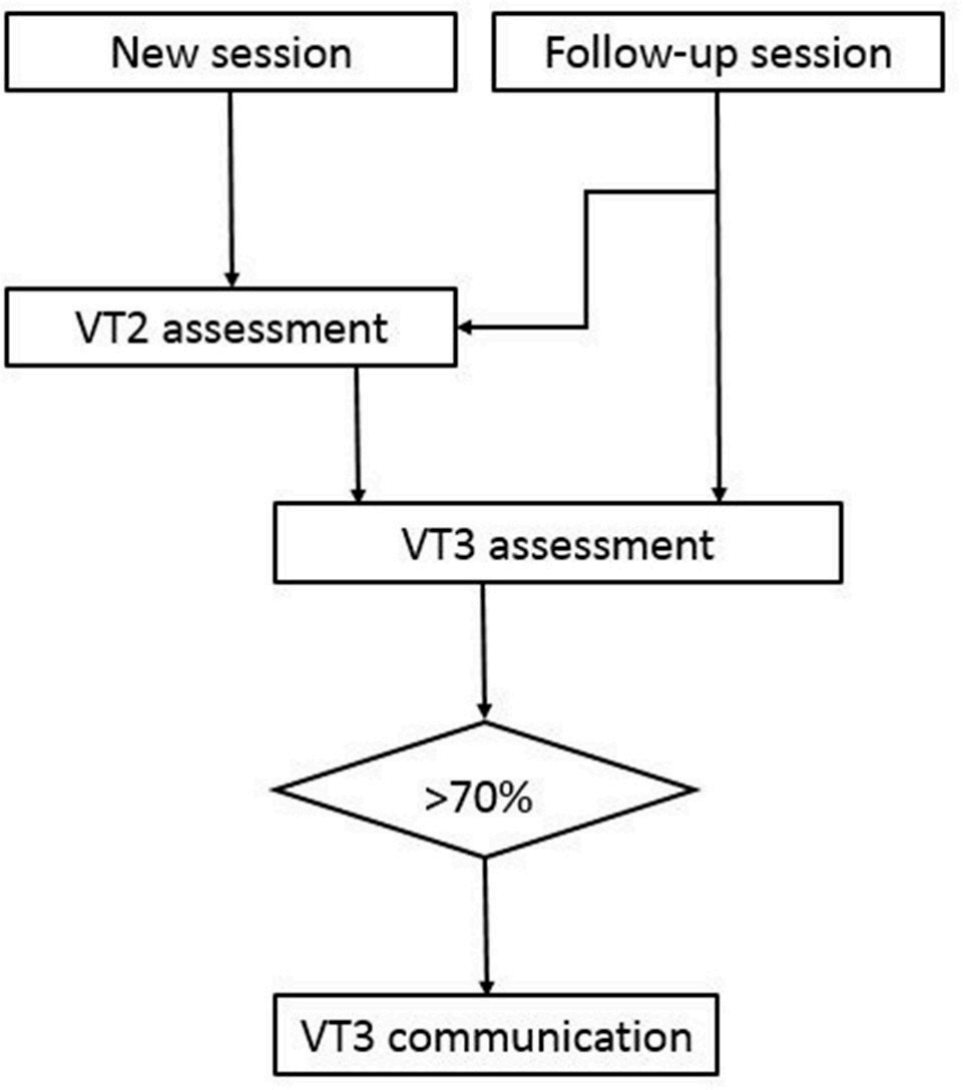
Experimental procedure. The first session for a new patient always starts with a VT2 assessment followed by a VT3 assessment. If the accuracy is above 70%, then VT3 communication was tested. Some follow-up sessions also began with VT2 assessment, whereas the other follow-up sessions instead began with a VT3 assessment to assess communication quickly.

Three paradigms were used: VT2 and VT3 assessment, and VT3 communication according the experimental procedure shown in Figure 1. VT2 uses two vibro-tactile stimulators that are fixed on the left and right wrists. Before each sequence of stimulations begins, the system verbally instructs the patient to silently count the stimuli on the target wrist. In the VT2 and VT3 assessment paradigms, the target wrist is selected pseudo-randomly, and each run has an equal number of left and right targets (15 each).

During stimulus presentation, the BCI system activates the vibro-tactile stimulation for 100 ms on the left or right wrist (also chosen pseudo-randomly), but the non-target wrist is stimulated more often (87.5%) than the target wrist to generate an oddball paradigm. The vibro-tactile stimulators are 3 cm long and 5 mm wide, and operate at 80 Hz. This paradigm is intended to generate a vibro-tactile P300 and other ERPs only when the target wrist is stimulated. In both VT3 modes, one additional stimulator is fixed on the back or shoulder as a distractor, which is active in 75% of stimulations. The other two stimulators are again fixed on the right and left wrist and each receive 12.5% of the stimuli. In all three modes, the BCI system instructs the subject to count the stimulations on either the left or right hand, which may elicit a P300 to the target hand. Each subject received 15 target stimulations and 7 × 15 non-target stimulations prior to a brief pause as an instruction to focus on the upcoming target wrist. During both the VT2 and VT3 assessment paradigms, each run lasted about 2.5 min. Each VT2 run contained 30 groups of eight stimuli (120 left, 120 right). Each VT3 run contains 30 groups of 8 stimuli (30 left, 30 right, 180 distractors).

The VT2 paradigm is usually performed first as an assessment run to see if the patient responds to the paradigm. The system will create ERPs from the assessment run and will also calculate the classification accuracy to show how well the target ERPs can be separated from the non-target ERPs. Then, a VT3 assessment run is performed to assess whether the patient is following commands in a paradigm with a distractor stimulus, and ERPs and the classification accuracies are calculated. These data are also used to calibrate the system on the subject specific EEG data. When the clinical conditions (i.e., alertness, heart rate, need of suction etc.) allowed it, we repeated the VT3 assessment only, in order to avoid prolonged sessions. All patients were assessed once in a day, except UWS 1, who was available for 4 sessions in a period of 2 months. This calibration information is used in further communication runs that allow the patient to say either YES (by counting the stimuli on the right hand) or NO (by counting the stimuli on the left hand). To limit the total recording time, we decided to conduct a VT3 communication run if a patient's accuracy was >70% in an assessment run (well above the 95% confidence interval with a binomial test that yields about 23% accuracy).

In the VT3 communication paradigm, the operator asks the subject a question just before each run begins, and the subject can answer either YES or NO by counting the stimuli on either the left or right hand. Thus, unlike the other two paradigms, the subject chose which wrist was the target. Ten customized and standardized questions to which the answers are known were used to evaluate system accuracy (e.g., Is your name Maria?; Is your son named Ricardo?,…). In the VT3 communication paradigm, one question can be answered after 120 stimuli, which requires 38 s. The system only selects YES or NO if the result is significant, and provides an “undetermined” response otherwise. The examiner then verbally repeated the answer displayed on the monitor.

### Data analysis and classification

Across all paradigms, we extracted data epochs of −100–600 ms around each stimulus and rejected trials in which the amplitude of the EEG signal exceeds ±100 μV. Each of these 700 ms data epochs was then sub-divided into 8 data segments of equal duration. We then created sub-averages for each of these data segments. Then, the data were classified using linear discriminant analysis (LDA), resulting in a classification accuracy ranging from 0 to 100% that describes how well the target vs. non-target data can be separated. The ratio of target to non-target stimuli is 1:7, resulting in a chance accuracy of 12.5%. (The classifier does not group the seven non-target stimuli together for classification purposes nor use a priori information about the target to improve accuracy.) In VT2 and VT3 mode, the data were randomly shuffled such that 50% of the data were used for training and 50% were used for testing to have independent training and testing data. This procedure was repeated 10 times.

A discriminable response was defined as a classification accuracy above 23% for VT2 and VT3 assessment (i.e., suggesting target vs. non-target ERPs could be discriminated). 23% is the 95% confidence interval tested with a binomial test. For VT3 communication testing, we defined communication as reliable if at least 70% of the questions were correctly answered.

**Figure 2:**
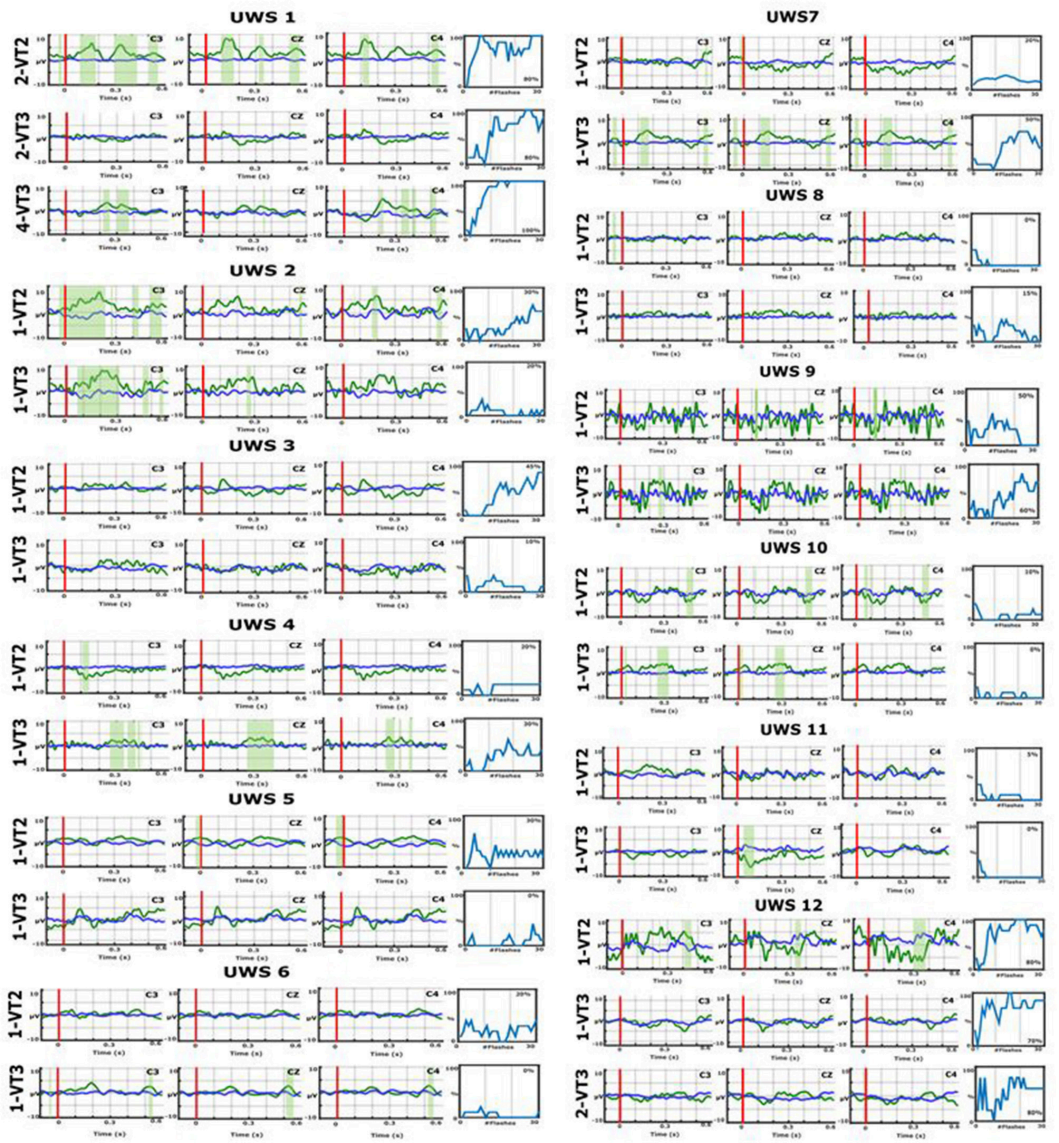
Event-related potentials (ERPs) over electrode sites C3, Cz, and C4 and BCI accuracies for VT2 and VT3 runs from all participants. The x-axes of the ERP plots present the time relative to stimulus onset, and the vertical red lines show stimulus onset at 0 ms. The blue lines reflect non-target ERPs, the green lines show target ERPs, and the green shaded areas show significant differences between these two traces. For example, in UWS1, the green shaded areas are most pronounced in the VT2 task, particularly over C3.The accuracy plots to the right of these ERPs show the resulting BCI accuracy. In each plot, the y-axis shows the % accuracy and the x-axis shows the number of trial groups (groups of eight stimuli) that were used to derive that accuracy.

In addition, we calculated the difference in ERPs between target and non-target stimuli during the VT2 and VT3 using a Kruskal-Wallis significance test using p<.05. Areas with significant differences between targets and non-targets are shaded green in Figure 2.

## Results

### Behavioral assessment

All the patients had at least 4 CRS-Rs within about 1 month (Median = 14.5 days, range = 7–34 days) before the first BCI study (8 patients had 5 CRS-Rs). The diagnosis was UWS before starting the study and remained the same for each BCI session.

Three patients could be assessed again a few weeks to a year post assessment. Two patients remained in a UWS after 1 year (UWS1 and UWS3), whereas one recovered signs of consciousness 15 days after the study (UWS12; visual fixation).

### BCI assessment

Eleven patients were seen for one or two runs in 1 day. The remaining patient (UWS1) was assessed for 2 runs per days on 4 different days (time between first and last day: 4 months).

The VT2/VT3 assessment and VT3 communication data are reported in Table 2. Figure 2 presents the ERPs from all patients over three central electrode sites, as well as the BCI classification accuracies. In the first session, a VT2 run was always performed to check the patient's ERPs elicited by the oddball task, then the VT3 run was performed to confirm active command-following ability (i.e., counting the target). In subsequent sessions, the VT2 run was sometimes skipped to go directly to VT3 assessment and communication testing. Each of the 12 UWS patients performed the VT2 and VT3 assessments at least once (between 1 and 4 runs for VT2, 1–7 runs for VT3).

**Table 2:** Median classification accuracies are shown for VT2 and VT3 assessment sessions for 12 UWS patients.

**Patient**	**Session #**	**VT2 assessment accuracy 4 instructions [%]**	**VT3 assessment accuracy 4 instructions [%]**	**VT3 Communication**
UWS1	1	100	0	-
		-	40	-
	2	25	20	-
		80	80	6/10 (4 wrong)
	3	60	0	-
		-	5	-
	4	-	100	7/10 (3 wrong)
UWS2	1	30	20	-
		-	0	-
UWS3	1	45	0	-
		-	10	-
UWS4	1	20	20	-
		-	30	-
UWS5	1	30	0	-
UWS6	1	20	0	-
UWS7	1	20	50	-
		-	0	-
UWS8	1	0	15	-
UWS9	1	50	30	-
		-	60	-
UWS10	1	10	0	-
UWS11	1	5	0	-
UWS12	1	80	70	-
		-	80	4/5 (1 undetermined)
Median		38.3	26.3	17/25 (7 wrong/1 undetermined)

*VT3 communication accuracy is presented as the number of questions answered correctly out of either 5 or 10 questions. For example, 4/ 1 /5 means that 4 answers out of 5 questions were given correctly and 1 answer was either undetermined or wrong. Runs (recordings within a session) are shown in different rows for a session (recordings on one day). A “-” shows that the paradigm or communication was not performed. The VT2 and VT3 assessment runs each last 2.5 min (4 instructions with 15 targets each). In VT3 communication, it takes 38 s to answer 1 question. ERPs of segments shaded in gray are shown in Figure 2*.

Using VT2, target vs. nontarget ERPs could be discriminated effectively in seven out of the 12 patients. Using VT3, 5 out of the 12 patients showed ERP differences suggesting command following. All the patients who showed performance above chance during VT3 assessment had a discriminable response to VT2.

Two patients (UWS1 and UWS12) reached a VT3 assessment accuracy >70%, allowing for communication testing. UWS1 reached 80% in the second session, run 2, and was able to answer 6 out of 10 questions correctly (60%). In session 4, run 1, UWS1 achieved 100% assessment accuracy and answered 7 out of 10 questions correctly (70%; the remaining answers were incorrect). UWS12 reached a VT3 accuracy of 80% in run 2 and could answer 4 out of 5 questions correctly (80%; 1 question was undetermined).

When looking at the ERPs of the patients who communicated (UWS1 and UWS12), UWS1 showed significant ERP differences for VT2 with an assessment accuracy of 80% in session 2 (see Figure 2). In the same session, the VT3 ERPs did not show significant differences in visual inspection, but the assessment accuracy was also 80%. In session 4, the VT3 assessment accuracy reached 100%, and the ERP showed significant differences.

In UWS12, the VT2 assessment accuracy reached 80% and a significant difference in the ERP could be observed. In VT3 run 1, the assessment accuracy was 70% and there was no clear difference between target and non-target ERPs based on visual inspection. In run 2, the VT3 accuracy increases to 80% and the ERP showed a difference.

Some additional patients showed differences in the ERPs. UWS7 showed no clear target vs. non-target differences for VT2, but showed stronger differences for VT3. In VT2 run 1, the mean accuracy was only 20%, but was 50% for the first VT3 run. Therefore, the VT3 assessment run was repeated, but accuracy declined and therefore communication was not tested. UWS4 showed a P300 response for the VT3 paradigm and achieved 30% accuracy. All other patients did not reach the significance threshold of 23% during the VT3 testing. Patient UWS2 showed a significant ERP on channel C3 in VT2 and VT3 mode, but the classification accuracy was not high enough to test communication. The same was true for UWS10. The other patients did not show significant differences in the ERPs.

## Discussion

The current study employed vibrotactile paradigms designed to elicit the P300 and other ERPs to evaluate covert command following and communication in UWS patients.

We reported that 41% of our patients showed signs of covert command following using the VT3 paradigm. In addition, 2 (out of 12; 16%) of the patients could establish reliable communication with the VT3 paradigm.

In the case of UWS1, 2 sessions were necessary to achieve a VT3 classification accuracy >70% to test for communication. In session 3, the accuracy decreased, which might suggest fluctuation in the patient's ability to follow commands, although the time in between sessions (i.e., about 2 weeks after session 2) prevents us from making strong assumptions. Finally, in session 4, the patient achieved a classification accuracy of 100%, allowing him to answer 7 out of 10 questions correctly. Interestingly, 88 days elapsed between sessions 1 and 4, and the patient immediately reached 100% in the last session in VT3 and could communicate. This result further highlights that performance can vary across sessions, and thus it is important quickly calibrate the system, assess the patient and proceed directly to communication mode if possible.

In the case of UWS12, communication could be tested after only 2 VT3 runs within a single session, leading to 4/5 correctly answered questions.

Our data appear to contradict what have been reported in previous literature on covert consciousness in DOC. We observe a higher number of patient showing signs of covert command following (41 vs. 17–20%). This could be due to the fact that we repeated the assessment, allowing us to take into account, at least partially, fluctuations in vigilance (Piarulli et al., 2016; Wannez et al., 2017). However, the 2 patients who could communicate both had a traumatic brain injury, consistent with previous literature on the effect of the etiology in covert cognitive abilities in severely brain injured population (Cruse et al., 2012). Further research should explore the relationships between BCI accuracy fluctuations and etiology, as well as exact diagnosis, time since injury and other factors.

The high variability across runs (as well as sessions) in the results highlights a significant challenge associated with this patient group. Patient UWS1 reached 80% VT3 assessment accuracy and could successfully communicate. In the next run, the accuracy was only 0%. UWS1 achieved 100% VT2 accuracy in the first run, which showed that he was able to execute the task correctly at that time, but the accuracy dropped to 0% in the subsequent VT3 test. In a second assessment performed 2 weeks later, he repeatedly achieved accuracy scores > 80%. Since UWS patients in Italy are admitted to intensive rehabilitation, the detection of command following in this clinically unresponsive patient did not directly affect the care plan.

If this neurophysiological finding had suggested a different prognostic scenario for this patient, it would not have been not correlated with outcome at 1 year, as the patient was still in the UWS. We cannot determine whether changes in medication or other treatment might have led to a different outcome, which is an interesting question for further study.

However, UWS12, who could communicate on the second run, started showing signs of consciousness 15 days after the BCI session, suggestive of MCS minus (i.e., visual fixation). Therefore, our data not only highlight the importance of repeated assessments to increase our understanding about the patient's profile and abilities; the data also show the importance of more research on the prognostic value of such tools in the clinical setting.

Table 3 summarizes results for UWS patients from the current study and from a previously published study on LIS/CLIS and healthy subjects (Guger et al., 2017b). Healthy subjects attained VT2 accuracies of 94% and VT3 accuracies of 88% (both in assessment mode) and a VT3 communication accuracy of 80%. With LIS and CLIS patients, we showed that 9 out of 12 are able to establish communication with VT3. Two of 12 UWS patients were able to communicate and the mean VT3 accuracy was 43.9%. LIS patients had a higher VT2 and VT3 accuracy when they communicated, but lower accuracies than healthy subjects. The CLIS patients that communicated attained VT3 accuracy higher than UWS patients. Among patients that could not communicate, VT2 and VT3 results were worst for UWS patients.

**Table 3:** VT2 and VT3 assessment accuracies, and VT3 communication accuracies, from healthy subjects and different patient groups (UWS, LIS, CLIS) from this study and a previous study (Guger et al., 2017a).

**Patient group**	**# Subjects**	**VT2 Assess [%]**	**VT3 Assess [%]**	**VT3 Comm. [%]**
Healthy	3	94	88	80
UWS	12	38.8	26.3	
UWS that communicated	2	69.0	43.9	75
UWS that did not communicate	10	23.0	15.7	-
LIS/CLIS	12	76.6	63.1	-
LIS that communicated	9	85.4	81.8	80
CLIS that communicated	2	60	85	80
LIS that did not communicate	3	56.7	24	-
CLIS that did not communicate	1	40	30	-

With healthy subjects and LIS/CLIS patients, the VT3 assessment paradigm appeared to be more difficult to perform than the VT2 assessment paradigm. Therefore, we suggest starting with VT2 to familiarize the patient with the easier approach, and then moving to VT3 within the limited time available.

### Limitations

This study would have benefited from additional patients. The study presents 12 patients with UWS resulting from different etiologies. The results showed that, regardless of the cause of the DOC, a considerable proportion of clinically unresponsive patients might show neurophysiological signs of command following. Due to the limited number of patients with each etiology, we cannot currently make strong claims about the relationship between etiology and command following. Further studies will explore this issue with more patients with different etiologies.

Similarly, we were only able to collect a limited amount of data from each patient. Communication was only tested if the accuracy was >70%, and communication was only tested in 1 or 2 sessions. In a previous study, the same VT2 and VT3 paradigms were used with 12 LIS/CLIS patients, and 9 of them could establish communication above an assessment accuracy threshold of 60% (Guger et al., 2017b). In addition, several patients were only assessed once. Hence, future work will assess the prospect of testing communication with lower assessment accuracies and collect data from more sessions. Training effects are also difficult to assess because UWS patients show fluctuations of awareness, and it is difficult to maintain a training schedule or study many sessions.

In UWS1, the first session was not promising, but sessions 2 and 4 showed that communication can be established. Furthermore, the communication testing could be improved by instructing the patient to say YES or NO to confirm that the patient understood the task correctly. In addition, more work should focus on defining the best threshold for assessing significance in such BCI systems. The CRS-R was done about 30 min before the VT2/VT3 testing and it lasts about 20 min, which might cause fatigue. Additional behavioral assessments in a shorter time-window, together with outcome data, may provide additional data to corroborate results from EEG assessments.

Another possible limitation is the lack of adequate somatosensory function. We did not test each patient's somatosensory capability, and thus cannot rule out the possibility that one or more patients would have exhibited better results with an auditory-based or motor imagery paradigm. The overall system used in this study can work with auditory evoked potentials and auditory-based motor imagery paradigms, but these were not tested here due to the very limited time available with each patient.

## Summary

Vibro-tactile P300 assessment using BCI technology provides a useful way to quickly test command following and establish YES/NO communication with some DOC patients. The paradigm provides a quick assessment that can be easily used to monitor fluctuations and to find the optimal times to communicate with these patients.

## Author contributions

RS, GM, and VL recorded the data. CG, AH, RO, WC, RX, and GE contributed to software development and/or software-based data analysis. CG, RS, VL, BA, JA, CC, and SL contributed to study design, scientific protocols, and review/analysis of results. RS, BA, and CG were primarily responsible for writing, and all authors discussed the results and implications.

### Conflict of interest statement

CG, AH, RX, and GE are employed by Guger Technologies OG, which is the company that built the mindBEAGLE system. CG, RO, WC, and GE are employed by g.tec medical engineering GmbH. The remaining authors declare that the research was conducted in the absence of any commercial or financial relationships that could be construed as a potential conflict of interest. The handling Editor declared a past co-authorship with the authors CG and BA.
